# Botanical Description of British Plants

**Published:** 1804-12-01

**Authors:** 


### *Botanical Description of British Plants.*

( Continued from pp. 361—372. )

60. Smyrnium. *S. olusatrum. Hipposetinum.*

*Ang.* Asisanders. Common Alexanders.

*Gen. Desc.* Petals keeled, tapering to a point. Fruit, egg-globular, bulging, angular.

*Spec. Desc. Stem leaves* by threes, on leaf-stalks, serrated. *Root leaves* by triple threes. *Sheaths* of leaves ragged, fringed. *Involucell.* v. short. Central florets male, other herm. Plant smooth, sickly pale green. Flowers green yellow, yellow. *Ditches, and rocks on the sea coast.* Bloss. May, June.

*Use.* Alexanders was formerly cultivated in our gardens, but it is now better supplied by celery, and consequently neglected. It is boiled and greedily eaten by sailors, who happen, on their return from long voyages, to land on the South-west corner of the Isle of Anglesea, where it grows in profusion.—*Pennant.*

61. Anethum. *A. fœniculum. Fœniculum dulce.*

*Ang.* Fennel. Finckle. Fennel-dill.

*Gen. Desc.* Petals entire, rolled inwards: Fruit lentil-shaped, compressed; small, scored, bordered.

*Spec. Desc. Leaves* many-div. hair-like. *Seeds* egg-oblong, tapering at each end, not bordered, three to five feet high; blue green. *Flowers* yellow. *Chalk Cliffs.* Bloss. July, August.

*Use.* This plant was highly esteemed by the ancients for promoting the secretion of milk, an opinion which the experience of some modern authors has tended to confirm. The seeds, though termed one of the four greater hot seeds, and supposed to be *stomachic* and *carminative,* are inferior in their effects to those of dill, anise, and carraway. The seeds used in medicine are usually imported from more southern countries: they give out their virtue very imperfectly in infusion, but by evaporation plentifully.—*Woodville.* They abound with an essential oil which is *carminative* and *diuretic,* but not heating.—*With.* The root, Alston says, may be called *alimentum medicamentosum;* and it was thought by Bergius to possess all the virtue of Ginséng, but it is now wholly disregarded in medicine: to the taste it is sweet, with very little aromatic warmth, and is said to be *pectoral* and *diuretic.* A simple distilled water is directed to be prepared from the fennel seeds.—*Woodville.* The leaves boiled are used in sauce for several kinds of fish, and they are eaten raw with pickled fish. The tender buds are useful in sallads. In Italy the stalks are blanched as a winter sallad.—*Withering.*

62. Carum. C. Carui. *Cuminum pratense. Caruon.*

*Ang.* Carraway.

*Gen. Desc.* Involucr. one leaf. Petals united, bent inwards, notched at the end. Fruit small, eliptical, bulging, roundish, scored.

*Spec. Desc.* Plant two to three feet high, smooth. *Leaves* doubly compound: *Leafits* in sixes, in a sort of whirl. *Involucr.* one to five leaves. *Umbel-sp.* nine to twelve. Florets all all fertile. Petals white, slightly tinged with red. *Meadows and pastures.* Bloss. May, June.

*Use.* The seeds are well known to have a pleasant spicy smell, and a warm aromatic taste, and are on this account used for various œconomical purposes. They are esteemed to be *carminative, cordial,* and *stomachic,* and are recommended in *dyspepsia, flatulencies,* and other symptoms attending hysterical and hypochondriacal disorders; they are likewise reported to be diuretic, and to promote the secretion of milk: they are less frequently employed than formerly.—*Woodville.* The seeds are used in cakes, and often put into bread, especially at Christmas-time. Incrusted with sugar, they are sold by confectioners as carraway-comfits; and they are often distilled along with spirituous liquors for the sake of the flavour and heat which they impart. These seeds were formerly recommended by Dioscorides to pale-faced girls, and, in more modern days, their use in that case is not forgotten. They are no despicable remedy in *tertian agues.* They abound with an essential oil which is *antispasmodic* and *carminative.—Withering.* Parkinson says, that the roots of this plant when young are a better esculent than parsnips. The tender leaves may be boiled with pot-herbs. Sheep, goats, and swine eat it: horses and cows are not fond of it.—*Ibid.*

63. Pimpinella. *P. magna.*

*Ang.* Great Burnet saxifrage, or anise.

*Gen. Desc.* Petals bent inwards. Styles upright. Summits nearly globular. Fruit small, egg-oblong, with five elevated ridges.

*Spec. Desc. Leaves* uniform, winged: *leafits* spear shaped, sometimes broader than they are long, irregularly serrated, as if besmeared with oil. *Leaf stalks* compressed. *Stem* two to three feet high. *Umbel-sp.* 14. *Pet.* white. *Hedges, woods.* Bloss. Aug. Sept.

*Use.* This species and the P. saxifraga, also a native of Britain, partake very nearly of the same qualities. In speaking of the latter, the medicinal of which however have no remarkable difference from the other, Dr. Woodville says, that the roots have an unpleasant smell and a hot pungent bitterish taste, whence chewing it has been recommended to relieve the *tooth ache:* its virtues are stated by Bergius to be *resolvent, diaphoretic, stomachic, diuretic.* It is recommended by several writers as a stomachic, and in all cases where pituitous humours are thought to prevail; and it is said by Hoffman to be an excellent *emmenagogue.* In the way of gargle it has been employed employed for dissolving viscid mucus, and to stimulate the tongue, when that organ becomes paralytic. It may be given in doses of a scruple in substance, or in infusion to two drachms.—*Woodville.* The root is so acrid as to burn the mouth like pepper; and, on account of its acrimony, it has been used to cleanse the skin from freckles. It is chewed to promote the secretion of saliva: and in Germany it is prescribed in asthma and dropsy. It affords a blue oil.—*Withering.*

64. Apium. *A. graveolens. A. palustre. Elcoselinum, Paludopium.*

*Ang.* Smallage. Parsley. *When cultivated,* Celery.

*Gen. Desc.* Involucr. one leaf. Petals equal. Fruit small, bulging, ribbed. Styles bent.

*Spec. Desc. Stem-leaves* wedge-shaped; root-leaves winged; *leafits* of three lobes serrated; *stem* smooth, shining, deeply furrowed. *Involucr.* often wanting. *Umbel-sp.* five to eleven; those of *umbellule* eleven to sixteen. *Petals* white. *Ditches and marshes.* Bloss. Aug.

*Use.* The root in its wild state, when it grows near water, is fetid, acrid and noxious, but when cultivated in dry ground, it loses these properties; and the root with the lower part of the leaf-stalk and stem, blanched by being covered with earth, are eaten raw, boiled in soups, or stewed; in this state of cultivation in the garden, it is known by the name of celery. It is said to be hurtful to those subject to nervous complaints: but it is certainly a good antiscorbutic. The seeds yield an essential oil. Sheep and goats eat it: cows are not fond of it: horses refuse it. —*Withering.*

65. Apium. *A. petroselinum. A. hortense. A. sativum.*

*Ang.* Common parsley. Garden parsley.

*Gen. Desc. As above.*

*Spec. Desc. Involucell.* very small; *root leaves* winged, by threes. *Stem-leaves* rise from the sheaths at joints; *leafits* cut into narrow line or segments, entire. *Umbel-sp.* ten. *Umbellule-sp.* twenty. *Petals* oval, white. Bloss. June, July.

*Use.* This plant is a native of Sardinia, but it has been so long cultivated in our gardens, and in such frequent use for culinary purposes, as to be more familiar to us than most of our indigenous plants. Both the roots and seeds are directed by the London College for medicinal use: the former have a sweetish taste, accompanied with a slight warmth or flavour, somewhat like that of a carrot: they are are said to be *aperient* and *diuretic,* and have been employed in apozems to relieve nephritic pains, and obstructions of urine. The seeds are in taste warmer and more aromatic than any other part of the plant, and also manifest a considerable bitterness: like those of many other umbelliferous plants, they possess a share of aromatic and carminative power, but this being inconsiderable, they are now but seldom employed. They have been used externally with advantage for destroying cutaneous insects in children. The bruised leaves have been successfully employed as a discutient poultice in various kinds of tumors; and it is said by *Lange (Misc. verit. Med. p.* 26,*)* that this application has succeeded in scirrhous tumours where cicuta and mercury had failed. Though parsley is in so common use at table, it is remarkable that facts have been adduced to prove that in some constitutions it occasions epilepsy, or at least in those subject to them, aggravates the fits: it has been supposed also to produce inflammation in the eyes.—*Woodville.*

PENTANDRIA. TRIGYNIA.

66. VIBURNUM. *V. Lantana.*

*Ang.* Wayfaring tree. Pliant mealy tree.

*Gen. Desc.* Cal. five div. superior. Bloss. five cleft; berry one celled, closed. Seed 1.

*Spec. Desc.* Leaves oval, serrated, veined, cottony underneath. *Flower leaves* coloured. *Bloss.* cloven, white. *Summits* united. *Berries* black. *Woods and hedges.* BLOSS. May.

*Use.* The berries are drying and astringent. The bark of the root is employed for the manufacture of bird-lime. —*Withering.*

67. SAMBUCUS. *S. ebulus. S. humilis.*

*Ang.* Dwarf elder. Wallwort. Danewort.

*Gen. Desc.* Cal. five-toothed. Bloss. regular, with five shallow clefts. Berry juicy, many-seeded, closed.

*Spec. Desc.* Tufts with three divisions. *Stipulæ* leaf-like. *Stem* herbaceous, very brittle, furrowed. *Leaves* winged; leafits spear shaped, serrated. *Cal.* segm. six, purple. *Bloss.* segm. pointed, white above, purple beneath. Anthers purple, properly ten, one on each side of each filament. *Berry* three celled, three seeded. *Hedges and road sides.* BLOSS. July.

*Use.* Every part of the plant has a faint disagreeable smell, resembling that of common elder, but stronger and more ungrateful: in the stomach its active power is greater. The root, which has a nauseous bitter taste, was formerly much employed in *dropsies.* A decoction of two drachms of it, or a small quantity of its expressed juice, promotes both the alvine and urinary discharges: if the decoction be prepared from the bark of the fresh root, its activity is so much increased, that it commonly proves both *emetic* and *cathartic.* The inner bark of the stalk, when recent, is equally powerful in evacuating the primæ viæ; and its effects as a *diuretic,* Dr. Brocklesby found to be very considerable. The berries, in their recent state, are said by Scopoli *(Flor. Carn.)* to be a gentle *cathartic,* though Haller did not find this effect. The seeds are also *diuretic,* and have been given with advantage in *dropsical* complaints: They also afford an oil, which Haller applied with success in painful affections of the joints. The leaves boiled in wine, and formed into a cataplasm, have been recommended in France as a *discutient* application to contusions and tumours.—*Woodville.* It is esteemed *diuretic* and *aperient.*—*Hill.* The roots are a powerful diuretic; a decoction of them has been found serviceable in the *dropsy.* —*Lightfoot.* It has the same medical properties with the *S. nigra (see next article),* but in some respects more violent, and therefore less manageable. One drachm and a half of the root is a strong purge. The berries give out a violet colour. The green leaves drive away mice from granaries: and the Silesians strew them where their pigs lie, under a persuasion that they prevent some of the diseases to which those animals are liable. Horses, cows, sheep, goats, and swine refuse it.—*Withering.*

68. Sambucus. *S. nigra. S. vulgaris. S. arborea.*

*Ang.* Elder. Common black elder.

*Gen. Desc. As above.*

*Spec. Desc.* Tufts five div. *leaflts* nearly egg-shaped, serrated. *Stem* tree-like. *Bloss.* white. *Anthers* yellow, one on each filament, arrow shaped. *Berries,* when ripe, deep black purple. *Seeds* 3. *Woods, damp hedges.* Bloss. Apr. May. *Note, Parsley-leaved elder is only a variety of this species.*

*Use.* This whole plant has an unpleasant narcotic smell, and some authors have represented it as unsafe to sleep under the shade of elder. The inner bark, flowers and berries are the parts used in medicine, and admitted into the pharmacopeias: the former is strongly *cathartic,* and on this account was much used by Sydenham and Boerhaave, who recommend it as an effectual hydragogue;

Sydenham

Sydenham directs three handfuls of it to be boiled in a quart of milk and water, till only a pint remains, of which one half is to be taken night and morning, and repeated for several days: it operates upwards and downwards, and on the evacuations produced, its efficacy depends. Boerhaave gave the expressed juice in doses from a drachm to half an ounce. In smaller doses it is said to be a useful aperient and deobstruent in various chronical disorders. The flowers have an agreeable flavour: in infusion when fresh they are gently *laxative;* when dry, they promote chiefly cuticular excretion, and are serviceable in *erysipelatous* and eruptive disorders. Externally they are used in fomentations and in the form of an ointment. The berries in taste are sweetish, and on expression yield a fine purple juice, which is a useful aperient and resolvent in recent colds and sundry chronical disorders, gently loosening the belly, and promoting urine and perspiration.—*Woodville, see Lewis, M. M.* This juice made into a *rob* is a safe and useful aperient.—*Lightfoot.* The inner green bark, which is an acrid *purgative,* is in smaller doses *diuretic,* and has done eminent service in glandular obstructions and dropsies: if sheep that have the *rot* be placed in a situation where they can get at the bark and the young shoots, they will quickly cure themselves. The leaves, like the bark, are *purgative,* but more nauseous. They are an ingredient in several cooling ointments. A decoction of the *flowers* taken internally is said to promote expectoration in pleurisies: externally, they are used in fomentations to ease pain and abate inflammation; they are used frequently to give a flavour to vinegar. The inner bark is an ingredient in the black dye. The berries are poisonous to poultry, and the flowers to turkies and peafowl. If turnips, cabbages, corn, or fruit-trees, (which are subject to blight from a variety of insects) be whipped with the green leaves and branches of elder, the insects will not attack them.—*Withering. (See Phil. Tr. lxii. p.* 348*).* The leaves bruised are sometimes applied outwardly in a cataplasm, in erysipelas and pleurisy, and are said to be very relaxing.—*Lightfoot.* The *wood* is hard, tough, and yellow; it is commonly made into skewers for butchers, tops for angling rods, and needles for weaving nets: it answers well for turning in the lathe. The pith being exceedingly light, is cut into balls used in electrical experiments.—*Withering.* Sheep eat it: horses, cows, and goats refuse it.—*Linn.* Others say cows are fond of it.

[ To be continued. ]

